# Research capacity strengthening in South Asia: based on the experience of South Asian Hub for Advocacy, Research and Education on Mental Health (SHARE)

**DOI:** 10.1017/gmh.2017.5

**Published:** 2017-05-16

**Authors:** M. Sharma, B. Razzaque

**Affiliations:** 1 Center for Chronic Conditions and Injuries, Public Health Foundation of India, New Delhi, India; 2 Institute of Psychiatry, WHO Collaborating Center for Research and Training in Mental Health, Pakistan

**Keywords:** Capacity-building outcomes, low- and middle-income countries, research capacity building, South Asia, teaching and learning

## Abstract

The South Asian Hub for Advocacy, Research and Education (SHARE) was a five-year National Institute of Mental Health (NIMH)-funded program that aimed to stimulate the research base for task-shifting mental health interventions to address the mental health treatment gap in low and middle-income countries. During its 5 years (2011–2016) SHARE made notable accomplishments, including providing 20 studentships for short courses and ten fellowships to conduct mentored study, developing a new humanitarian research training course, implementing distance learning courses, creating an online repository of training materials, creation of a network of public health researchers at different career stages in South Asia, strengthening of partnerships amongst institutions of SHARE network and supporting its member's to produce peer reviewed publications. Furthermore, additional research capacity building and research grants leveraged on SHARE network were secured. The salient lessons learned in the 5-year program were that research capacity-building opportunities need to be tailored to the local context, as SHARE sought to develop and support courses that can build the capacities in specific areas identified as weak in the South Asian region. Mentoring was recognized as a critical component for which innovative and effective models of mentoring in the region need to be developed. Diverse platforms and mediums ought to be utilized to deliver the research training programs. Finally, research capacity-building program requires collaborative efforts of multiple stakeholders working locally, nationally and globally to attain the maximum impact in a region.

## Introduction

Mental health disorders are highly prevalent, with 14% of the global burden of disease attributed to neuropsychiatric disorders (Prince *et al.* 2007). It has been estimated that more than 75% of those identified with serious anxiety, mood, impulse control or substance use disorders in low- and middle-income countries (LMICs) received no care (Demyttenaere *et al.* 2004), and mental health care and mental health research is a low priority area. The paucity of research outputs from LMICs has been documented by various studies from time to time (Patel & Sumathipala, 2001; Patel & Kim, 2007; De Jesus Mari *et al.* 2009; Thornicroft *et al.* 2012). In response to this low contribution to global mental health research by LMICs, the Mental Health Research Mapping Project was initiated by the Global Forum for Health Research and the World Health Organization in 2005 with the goal of describing the current research capacity, resources, and agendas in LMICs. The survey showed a severe scarcity of mental health research resources, inequitable distribution and inefficient utilization of these resources in the surveyed countries. This survey found that most researchers and publications were concentrated in 10% of the 114 LMICs (Razzouk *et al.* 2010). Low publication rates from LMICs were reported to be due to many disadvantages faced by researchers such as lack of human resources, lack of access to journals and databases, limited research fellowships and funding (Sharan *et al.* 2009; Razzouk *et al.* 2010).

The focus of research studies also differs across the LMICs and high-income countries (HICs). Most research studies conducted in LMICs (up to 80%) were found to be on epidemiology, social, psychological and clinical aspects, while most intervention trials (up to 87%) were conducted in HICs (Sharan *et al.* 2009).

Mental health research can play an important role in addressing the treatment gap, by increasing our understanding of unmet mental health needs, effective resource allocation, policy and planning, establishing and scaling up of services. The fact that LMICs have failed to take on the role of a lead player in conducting and publishing mental health research has further contributed to this dire situation of the enormity of disease burden and treatment gap.

## Mental health research in South Asia

South Asian region is one of the most populous, poorest and least developed regions in the world (UNDP report 2010). A key challenge in mental health research in South Asia is inadequate capacity and resources to conduct research. The mental health research mapping project concluded that the South Asian region suffers from extremely inadequate mental health research resources in terms of both financial support and professional support (e.g. involvement in research networks, training in research methodology, skills in writing papers) (Sharan *et al*. 2007). It reported meager research outputs from South Asia, as only 36% of respondents to the survey reported more than five publications in the 5 years prior to the survey. The mapping project documented several barriers to publishing research including limited access to capacity-building opportunities, lack of research networks and lack of expertise in the language and style needed for publication in indexed journals. It was reported that there is a little interface between research and policy (Sharan *et al.* 2007). Additionally, in South Asian countries, most training in mental health happens in medical schools where the focus is mainly training in clinical skills. Mental health professionals receive little training in research, if at all, and it happens as a part of postgraduate medical trainings where they are required to work on small-scale projects. Brain drain amongst highly trained health professionals to developed countries is common (Mullan, 2005).

## Barriers in capacity building in South Asia

The barriers that impede the career growth and skills enhancement of researchers in South Asia are many fold.

Most countries in the region have limited (or non-existent in few cases) opportunities to build research capacity. The distribution of training resources is skewed, as most training institutes are concentrated in few countries (India, Sri Lanka and Pakistan).

In general, most academic institutes in the region lack adequate financial support, opportunities and infrastructure to support research and training opportunities for mental health researchers. Lack of access to research networks for skills enhancement and mentoring are other factors that limit research capacity building across the region.

A barrier at the level of institutes is the lack of experience to lead or mentor mental health research projects, as there is a general lack of a research culture in the health sector. The lack of mentorship to guide and support young or early career researchers impedes their professional progress or ability to formulate clear career goals in the field of research.

Furthermore, the research institutes often lack the capacity to utilize or translate their research findings into policy as a result of limited collaborations with policy makers or civil society. Across the region, the level of competition for limited funding opportunities remains high. Researchers, especially those at entry level or middle career level, often lack the necessary guidance to develop strong proposals for research grants.

## About SHARE

South Asian Hub for Advocacy, Research and Education (SHARE) on Mental Health was founded as a collaborative network of academic, research, and policy institutions spread in six South Asian countries, such as India, Pakistan, Nepal, Sri Lanka, Afghanistan and Bangladesh (website http://www.sharementalhealth.org). SHARE, one of the collaborative hubs for international research on mental health funded by National Institute of Mental Health (NIMH), was established with the primary aim to create a network of institutional partners in South Asia and to carry out research that answers policy relevant questions related to reducing the treatment gap for mental disorders in the South Asian region, through a concerted program of research on task-shifting and research capacity building. The core activities of SHARE are briefly discussed below.

### Administrative core

SHARE administrative Core had the responsibility of overall governance of the project. SHARE had two administrative cores, one each in India and Pakistan. Two Administrative cores were established for pragmatic purposes due to the troubled political climate prevailing in the region. The admin core also facilitated the collaborations for capacity building and research between SHARE network partners.

### Research core

The aim of SHARE research core was to develop an innovative, feasible, effective and sustainable community-based approach for the delivery of an established psychological treatment to reduce the burden of depression in mothers in South Asia. The research teams worked in collaboration with government maternal health programs in sites in India and Pakistan.

### Capacity building core

The overall goal of the SHARE research capacity-building component was to strengthen the capacity of partner institutions and researchers associated with these institutes, to generate, communicate and utilize mental health research with the goal of reducing the treatment gap for mental disorders in the region.

By implementing these core activities, by building a network of diverse institutions, and by involving the participation of policy makers, SHARE aimed to implement a coherent program to reduce the massive treatment gap in the region.

## SHARE research capacity-building activities

The research capacity-building component of SHARE was designed to overcome some of the barriers to research capacity building in South Asia, by building a multi-disciplinary network of mental health researchers at different career levels across its partner countries. All its research capacity-building activities aimed to maximize individual and institutional research capacities thereby enhancing access to training in the relevant research skills, promoting networking and collaboration between partner institutions and researchers, and facilitating knowledge translation and exchange. Table 1 has a brief description of capacity-building activities of SHARE.

**Table 1:** Research capacity-building activities of SHARE (2012–2017)

Research capacity-building activity	Objective, description	Recipient of opportunity	Outcome
Studentships	Objective: To build capacity in young and mid-level researchers from the region through participation in annual short courses conducted by SHARE institutions	The applicants and awardees for this program were mainly early career professionals associated with SHARE network institutes, with at least a graduate degree in a health science-related discipline and an interest in working in mental health	Seventeen researchers associated with SHARE network successfully completed the courses offered by SHARE network Three South Asia-based courses were offered: Leadership in mental health Development and Evaluation of Complex interventions Qualitative methods for health research and medical discourse
	Studentship awardees were selected on competitive basis		
Fellowships	Objective: To build capacity in researchers associated with SHARE network, to carry out mentored research in a topic of their choice relevant to the overall goals of SHARE.	Fellows were early/middle career professionals associated with SHARE institutes, with at least a graduate degree in a health science-related discipline, 2 years of work experience in public health in South Asia, and had defined interest in building research career in mental health in South Asia	Seven researchers successfully completed their study under mentorship of a senior SHARE researcher Three Publications in scientific journals (weblink http://sharementalhealth.org/index.php?option=com_content&view=article&id=26&Itemid=645) Two publications are in preparation Two fellows won grants to scale up their work of fellowship study in their region
	Mentors were the senior/middle level researchers associated with SHARE network, who had the expertise and experience of working in mental health in South Asia.		
	A list of mentors was prepared and the fellows found a match by sending request to the mentors.		
	Fellows were selected on competitive basis		
Mental health services research in humanitarian context’	Objective: To produce a cohort of skilled researchers and practitioners able to make a significant contribution to mental health research in a humanitarian context in the South Asia region. This course was developed by SHARE partners, University of Liverpool, Johns Hopkins University, Institute of Psychiatry Pakistan. It is a 2 years long course, spread over four module – weekly classroom-based teaching every 6 months delivered in Pakistan Salient features: Research-led, thereby evolution of research projects drove the course Enquiry-based, engaging with course content through participatory train Cascade training model – Training of Trainers ToT model Online learning environment to support of the participants in between modules	The applicants and awardees for this program were mainly early career professionals associated with SHARE network One cohort – comprising of 8 participants. Six from Pakistan, one from Sri Lanka and one from Nepal	Classroom-based modules delivered All participants have successfully completed qualitative assessments in a range of settings: Swat Valley, Peshawar in Pakistan, Islamabad/Pindi in Pakistan, Northern Sri Lanka in an area only recently made accessible again following conclusion of the war, Chitwan district of Nepal All participants have completed work on reports for module 1,2 and 3. Work underway on their trail designs One publication in scientific paper Two other publications are in preparation Three additional small grants for support of the SHARE course ‘Mental Health services research in Humanitarian context’ course Local capacity building – 30 people trained in needs assessment in humanitarian context according to DIME in workshops and seminar
Distance learning course ‘Implementation science for Public Health Interventions’	Objective: The course aimed to provide a systematic framework for implementation and evaluation of public health interventions.	Public health professionals working in LMICs, with a year of work experience in implementation project in health/development sector.	Twelve public health professionals working in LMIC s completed the first batch of course in Oct. 2014 Course team has modified the course (based on feedback from first batch) and revised course is expected to be live by first quarter of current year available to participants around the world, who are eligible and interested in the course
	This course was developed in a distance learning format, with combination of online audio and video lectures, interactive discussion board, electronically submitted assignments and recommended readings	First batch had 14 participants associated with NIH collaborative hubs only
Online repository ‘Super course lectures on Global mental health’	Objective: To create an online repository of lectures on mental health research that is available in open-access format and can be incorporated into any capacity-building program around the world.	Open and free access to researchers around the world	Eleven lectures have been uploaded on the course website (Course weblink http://www.pitt.edu/~super1/GMHS/index.htm Some more lectures are in the process of being uploaded
	This online repository is hosted as a part of supercourse library of lectures on global health. SHARE and another NIH hub ReDeAmericas are working in collaboration to develop the lectures for mental health repository		
Online discussion forum	Objective: To provide mental health researchers from SHARE institutions with support, guidance and the opportunity for networking with peers and mentors.	Access was restricted to members associated with SHARE network institutes	This forum was closed due to the non-participation by the members
	The membership of this online forum was restricted to only those who are associated (working) with the SHARE network institutes.
	The forum was moderated by a senior/middle career researcher from the SHARE network on a monthly rota basis

From the outset SHARE partners were cognizant of the importance of addressing the local sociocultural contexts in each country. Keeping this principle in mind all the researchers and fellows associated with SHARE were encouraged and guided to conduct their research by adapting the universally applicable research tools and methodologies to the local context.

Another important aspect taken into consideration was the inequitable distribution of training opportunities and resources to conduct research across the South Asian region. To address this, a central aspect of the network activities included facilitating the formation of regional networks to support the individual researchers and institutes working in the mental health field in South Asia.

SHARE research capacity-building activities included both developing new activities that sought to address the identifiable gaps in research capacity-building opportunities, as well as supporting the existing activities. All research capacity-building initiatives supported by SHARE were a product of collaborative partnerships between SHARE network partners in South Asia and beyond. For instance, ‘Leadership in Mental Health’, a training course being conducted since 2008, involved four SHARE partners (London School of Hygiene and Tropical Medicine UK and Sangath, Public Health Foundation of India and Schizophrenia Research Foundation in India), SHARE offered this course to a wider audience in the region, with a special emphasis on increasing the participation of researchers working in region with limited opportunities for research training.

SHARE fellowship program and Online Mentoring Forum were programs designed in response to the challenge faced by early and middle career researchers, i.e. individuals in need of mentoring, networking and career guidance opportunities. The Fellowship project had a unique aspect as it entailed provision of close mentoring of early career researchers who either lacked support from their institutes or within their region to conduct research. All the SHARE fellows were matched with senior researchers from SHARE network, for provision of close supervision and guidance both on conducting and dissemination of study results. Online mentoring program involved formation of an online community of researchers (both junior and senior) to facilitate exchange of ideas, networking and career guidance for junior researchers by the experts in the group.

SHARE designed and rolled out two short capacity-building courses to meet the existing demands in the region. In the more recent years, South Asian countries have been afflicted by both natural and man-made disasters. It was detected that interventions provided to the affected population were neither tailored to the sociocultural context, nor had they shown significant impact. A need then arose to train and disseminate the principles of carrying out ethically sound scientific humanitarian research imbedded in socio-contextual setting. For this purpose, SHARE established collaborative partnership with international institutes to develop and implement new capacity-building initiatives in South Asia. One such initiative was a 2-year course on conducting mental health research in humanitarian context, this was as a collaborative initiative with international experts from Johns Hopkins University and University of Liverpool. The course also offered mentor ship to the participants, which entailed guidance and support with the research projects, and adaptation of interventions in accordance with the sociocultural setting.

The Distance learning course of SHARE on ‘Implementation science for Public Health’ was designed to meet the increasing demand for low cost and virtual Implementation science courses for researchers in LMICs. This course was developed and implemented by SHARE administrative core at the Institute Public Health Foundation of India and experts from the University of North Carolina.

SHARE collaborated actively with other NIMH-funded hubs to develop capacity-building programs for LMICs. Capacity-building efforts of all five NIMH-funded hubs are described in an article published jointly by representative members of all hubs (Pilowsky *et al.* 2016). SHARE and REDEAMERICAS (Latin American hub) collaborated to establish an open access library of super course lectures on global mental health. Senior researchers have conducted webinars on several pertinent topics (such as applying for competitive grants, conducting research in humanitarian context, maternal depression), for young fellows and researchers associated with the five hubs.

The SHARE research capacity-building initiatives utilized various platforms to implement its research capacity-building programs. The distance-learning course ‘Implementation science for Public Health interventions’ was developed an online course that can be offered on virtual platform to researchers anywhere in the world. Additionally, a virtual repository of supercourse lectures on global mental health was created to be accessed at no cost. The Humanitarian course was based on a hybrid model that utilized both onsite training and virtual technology. Most SHARE fellows sought guidance from their mentor using online mediums.

The senior management group took onboard the mandate to provide career development guidance to the researchers, fellows and associates of SHARE. SHARE senior team members remained involved in providing guidance to young researchers in applying for research grants, for publication of their work in high-impact regional and international journals, and communication of research findings to diverse stakeholders with an aim of translating them into policy and practice.

The core capacity-building team has regularly conducted annual career tracking of all awardees of capacity-building programs (Studentships, fellowships, humanitarian course, etc.). All the capacity-building beneficiaries were asked to keep the core capacity-building team updated about their current professional status, new professional developments and need for career-related guidance.

## Strengths, success stories and limitations

The pivot of SHARE research capacity-building activities remained creating a cadre of mental health researchers in South Asia, contributing to fulfillment of the training needs of researchers at different stages of their careers, establishing and nurturing networks amongst institutions across the region, and fostering the provision of support to researchers working under varied resource settings. The inter-disciplinary strengths of the investigators and prior expertise and planned commitment to mentoring remained major strengths of SHARE research capacity building.

The research grants offered numerous research skills enhancement opportunities to its early career researchers. The senior investigators associated with the research core purposefully involved the young researchers in all academic publications of research trial. All SHARE fellows were encouraged to disseminate their findings to a wider audience at research symposiums, conferences and public engagement events. In the first 4 years of the project, the project researchers and fellows published ten papers in peer-reviewed journals. Details of all publications and dissemination outputs of SHARE can be found on SHARE website (weblink: http://sharementalhealth.org/index.php?option=com_content&view=article&id=26&Itemid=645).

SHARE team members have also been successful in applying for research and Capacity-building grants leveraged on the ongoing SHARE work. In order to address low levels of participation of junior researchers in grant writing, junior researchers of team were involved and mentored by the senior researchers in the grant writing and application process. The details of research grants can be found at: http://sharementalhealth.org/index.php?option=com_content&view=article&id=71&Itemid=715.

The SHARE Senior Management team remained committed to providing support to the students and fellows associated with the network to help them build a career in mental health research. Three of SHARE fellowship awardees from India, Nepal and Sri Lanka have been successful in winning grants to expand their fellowship research, after the completion of their SHARE supported fellowship study. Six of the researchers and fellows progressed to attain higher degrees (Doctorate, Post doctorate). Of these, four-doctorate studies were embedded in the ongoing SHARE research trial, and the senior researchers continued to provide close supervision and guidance to these candidates.

Some researchers utilized their partnerships outside the SHARE network, to facilitate the supervision of young researchers based in regions with limited capacity and opportunities to support research. For instance, one of the SHARE PIs helped in finding a mentor for a SHARE fellow from Afghanistan by assisting the trainee in matching with one of his associates based in the same region. Feedback from studentship awardees has further fortified the SHARE success, as they reported that participation in the SHARE short courses helped young researchers build confidence, be orientated to mental health research and to form networks with mental health researchers from different backgrounds and regions.

The newly developed humanitarian course proved to be a success as the candidates learned principles of scientifically tested, widely used research methodology, applied the principles to research projects based in humanitarian settings, and learned to adapt interventions to cultural settings. Despite the success, a 2-year longitudinal course duration proved to be a limitation as the length of the course compromised sustainability of the course cohort and two dropouts were reported.

The SHARE program faced certain administrative challenges in the implementation of its research and capacity-building activities. The South Asian region is a diverse region with relationships between countries marked by changing political and economic situation. The core capacity-building team of SHARE faced challenges resulting from cross-border tensions that made it hard for researchers from certain regions to participate in various capacity-building activities and collaborative research programs. Moreover, different sets of administrative regulations across the institutes in the region led to complications and delays in starting or sustaining certain capacity-building activities.

Despite the best efforts to provide equitable research capacity-building opportunities to candidates from all six countries in the region, more opportunities remained skewed to settings where the infrastructure already existed. Both the SHARE studentships and fellowships candidates had maximum participation from countries such as India and Pakistan. Researchers from settings that lack infrastructure and support faced significant barriers to leverage the training opportunities. For instance, a researcher from Afghanistan (a country that lacks research capacity-building opportunities all together) faced significant barriers in terms of sustaining contact with mentors and finding a local mentor, due to the logistical issues.

Keeping all these issues under consideration, SHARE developed web-based programs to make the courses more accessible across the region. Despite this provision some countries in the region lacked infrastructural support such as Internet connection thereby making web-based solutions difficult to implement. Language remained another barrier, especially for researchers who were not fluent in English as the training courses were available only in English. Additionally, lack of fluency in English also impacted the ability of some of the researchers to produce quality publication or participate in global dissemination events.

SHARE faced certain challenges in implementing its mentoring-based programs (i.e. online mentoring program and the fellowship program). The online mentoring forum had to be discontinued due to low participation by the members. This closed online learning forum was initiated by SHARE, with the aim of providing a platform for discussion, mentoring, networking and exchange of ideas. Being developed as a moderated forum, it required one member (either a middle level or senior researcher) to spend 2 hours a week initially to moderate the discussions. This posed a barrier, as most senior researchers were unable to commit time to facilitate the discussions on forum. It was also noticed that participants stopped participating in the forum as they reported a preference for an individual interaction instead of an online group interaction to seek career related guidance.

Another significant challenge faced in research capacity building was associated with matching the early career fellows with mentoring and guidance opportunities, an example was that of a SHARE fellow based in Afghanistan (i.e. a country that lacks trained researchers) who faced great difficulty in finding a mentor for his fellowship study. In addition, poor internet connectivity also posed a significant issue to connect with mentors living abroad.

Moreover, most fellows required intense and regular supervision, which became difficult to provide by most of senior researchers associated with SHARE network due to their busy schedules. The SHARE team dealt with this by assigning two more mentors, both middle career researchers who lived in the same geographical zone and had some experience of conducting research in the region.

## Discussion

The research capacity-building activities of SHARE were aimed at maximizing individual and institutional research capacity development by enhancing access to training in the relevant research skills, promoting networking and collaboration between partner institutions and researchers, there by facilitating knowledge transference and exchange. SHARE instituted the aforementioned capacity-building activities in order to train a cadre of researchers in South Asia and to empower them in carrying out mental health research and dissemination of the research findings, thereby decreasing the treatment gap for mental health conditions. Formation of peer network of researchers was the pivot of success of the SHARE program. At the end of this 5-year program, SHARE has effectively managed to roll out 5 capacity-building programs (Table 1), all aiming at orientating the early and middle career researchers to mental health research and equipping them with the necessary skill set needed to carry out scientific research.

Lack of mentorship and professional guidance was highlighted as a barrier to evidence-based research in the region. Recognizing this, SHARE tried to build a collaboration of partners by assembling and nurturing a network of mentors from diverse disciplines to support early and middle career researchers. South Asia (and in other LMICs) has limited and overburdened expert researcher resources. In this region, mentoring and providing career guidance has historically remained a voluntary activity in most institutional settings. The SHARE capacity-building team realized that the model based on one–one mentoring by senior researchers may not be sustainable to build research capacity in a region where there are few trained researchers; therefore, the SHARE team endeavored to form a network of mentors at different career stages. Thus, at the end of its 5 years, SHARE has successfully brought together a bank of researchers with varied experience and areas of expertise, to provide guidance or career advice to young or early career researchers.

The research capacity building employed all available avenues of learning including various modes of delivery (onsite, online and hybrid training methodology) in almost all SHARE training programs, this experience has clearly demonstrated that the creative use of the web-based technologies helps not only to transcend boundaries, but also remains a resource-efficient modality of training in a resource-stricken environment such as South Asia. The team created and operated a distance-learning program intended to leverage research capacity building of a wider audience, in collaboration with academic partners in India, Sri Lanka and Bangladesh. SHARE co-created an online library hosted on global forums to help in wider dissemination of mental health resources.

Furthermore, SHARE partners, being cognizant of the research needs of the regions, worked to ensure sustainable models of research capacity building. A momentous success of SHARE has been achievement of sustainability and accessibility of training opportunities in the region. SHARE accomplished this by developing various short distance-based virtual platforms for short courses, and by offering ‘classic’ lectures on mental health in open source formats for the global community. SHARE experts helped develop the research capacity-building activities specifically relevant to the region. Mental Health Services Research in Humanitarian contexts, was a new course developed to address the ethically grounded scientific research needs in the region. The strength of the course was the cascade model, thereby ensuring that master trainers are trained in the region to ensure the momentum of further trainings continues in the region. This training model will ensure further dissemination of research capacity-building activities.

An important lesson learnt is that to build a sustainable model of research capacity building in a region with inadequate and inequitable resources, it is imperative to involve researchers at different career stages and regional institutes with complementary roles and expertise. The SHARE network consisted of an inter-disciplinary team of members that helped in the cross-germination of training across its institutes. For instance, the representative of Nepal mental Health foundation (civil society NGO in Nepal) is one of the trainers for the course ‘leadership in mental health’ that is offered by Sangath (research institute and NGO in India). A senior investigator from Sangath mentored a fellow from the Nepal Mental Health Foundation to conduct study in Nepal. Some of SHARE partner institutes (such as the Public Health Foundation of India, Institute of Psychiatry in Pakistan, Institute for Research and Development in Sri Lanka) work closely with the Ministry of Health in their respective countries. This gave an opportunity to SHARE core team to initiate a dialog with the government agencies and decision makers, and conduct training programs and dissemination programs for the relevant government bodies.

SHARE core capacity-building team is working with the core teams of other four hubs, to plan for the continuity of capacity-building programs in LMICs, beyond the lifetime of all hubs (Pilowsky *et al*. 2016). In the future, the SHARE PIs and senior management team would continue to support the regional network in South Asia. SHARE facilitated creation of a platform for institutes working toward a common goal of reduction of the treatment gap in South Asia, to come together to talk about their unique experiences and recognize each other strengths. We expect the network collaborations to strengthen and expand the research capacity in South Asia in coming years.

## Conclusion

SHARE research capacity-building initiatives were conceptualized keeping in view limited research capacity, low number of scientific outputs and various barriers that exist to conducting and disseminating research findings and the huge mental health disease burden in South Asia. Upon completion of the grant period, SHARE leaves behind the legacy of energetic network of institutes in South Asia, working together on mental health research and research capacity building. The SHARE core team hopes that the cross-institutional mentoring and collaborations for research and training would continue beyond the life of SHARE. The salient outcomes of this 5-year collaboration have been a significant number of people trained in conducting research, research publications in leading journals, new research grants procured and finally partnerships created not only in the region, but also across the world with an aim to work in a concerted effort toward training in research capacity building. SHARE will continue to monitor and track the career development of the researchers on a yearly basis. The project hopes to continue to nurture these partnerships as this culture of collaboration has proven to be integral to the cause of bridging the treatment gap in the region.
